# Digestion Versus Drugs in the Treatment of Pulmonary Tuberculosis

**Published:** 1898-02

**Authors:** 


### Digestion versus Drugs in the Treatment of Pulmonary Tuberculosis.

Fisk (Transactions of the American Climatological Association, 1897) again inveighs in his forcible manner against polypharmacy and useless drug-giving in cases of phthisis. He believes that digestion should be protected at all hazards. Frequently he has seen patients taking many drugs and failing rapidly, because they had no appetite and could not digest what they ate, improve rapidly when the digestive tract was cleared by the use of calomel.

By digestion he means the ability to eat, to assimilate, and to eliminate—in short, to nourish properly.—*University Med. Magazine.*

